# Intervenciones para mejorar la experiencia de familia/acompañantes durante la espera quirúrgica: Una revisión sistemática

**DOI:** 10.23938/ASSN.1094

**Published:** 2025-02-26

**Authors:** María José Moreno, Elena Martín-Gómez, Mónica Vázquez-Calatayud

**Affiliations:** 1 Clínica Universidad de Navarra Pamplona España; 2 Universidad de Navarra Grupo de Investigación Innovación para un Cuidado Centrado en la Persona (ICCP-UNAV) Pamplona España; 3 Universidad de Navarra Facultad de Enfermería Pamplona España; 4 Instituto de Investigación Sanitaria de Navarra (IdisNA) Pamplona España

**Keywords:** Acompañantes de Pacientes, Periodo Perioperatorio, Ansiedad, Intervención de Enfermería, Revisión Sistemática, Accompanying Family Members, Perioperative Period, Anxiety, Nursing Intervention, Systematic Review

## Abstract

**Fundamento:**

El papel de familia/acompañantes es clave en la adaptación postquirúrgica del paciente, favoreciendo su recuperación y actitud positiva hacia el hospital. El objetivo fue identificar intervenciones efectivas que mejoren la experiencia de familia/acompañantes durante la espera quirúrgica.

**Métodos:**

Revisión sistemática siguiendo las recomendaciones PRISMA. Se aplicó la estrategia de búsqueda adaptada a las bases de datos PubMed, CINAHL, SCOPUS, PsycINFO y Cochrane, de artículos en español o inglés publicados entre enero de 2013 y diciembre de 2023. Se obtuvieron los siguientes datos de los estudios: país, año, diseño, tamaño de muestra, objetivo, características de las intervenciones, instrumentos para medir su efectividad y principales resultados obtenidos. Su calidad metodológica se evaluó mediante las herramientas del *Joanna Briggs Institute*.

**Resultados:**

En los ocho estudios incluidos se identificaron tres tipos principales de intervenciones, basadas en la comunicación estructurada, telemática y continuada. Las intervenciones fueron heterogéneas respecto a componentes comunes como proveedor, momento, lugar, formato, frecuencia y duración, y no todos los estudios los especificaban. Destacó la efectividad de la comunicación telemática mediante vídeos explicativos de al menos 20 minutos en la sala de espera, los cuales redujeron significativamente la ansiedad familiar, medida con la escala STAI.

**Conclusiones:**

Los hallazgos destacan la escasez de intervenciones diseñadas para mejorar la experiencia de familias/acompañantes durante la espera quirúrgica, y la necesidad de estandarizar intervenciones e instrumentos de medida de resultados, para optimizar el apoyo emocional brindado y contribuir a una mejora integral en la experiencia y bienestar familiar en este contexto.

## Abstract (en)

**Background:**

Family members and companions play a crucial role in supporting a patient’s postoperative recovery, fostering a positive attitude toward the hospital, and promoting overall well-being. This study aimed to identify effective interventions designed to enhance the experience of family members and companions during surgical waiting periods.

**Methods:**

A systematic review was conducted following PRISMA guidelines. A comprehensive search was performed in the PubMed, CINAHL, SCOPUS, PsycINFO, and Cochrane databases for articles published in Spanish or English between January 2013 and December 2023. Data extracted from the studies included: country of origin, publication year, study design, sample size, study objective, characteristics of the interventions, tools used to measure effectiveness, and key findings. Methodological quality was assessed using Joanna Briggs Institute critical appraisal tools.

**Results:**

Eight studies were included, revealing three main types of interventions: structured communication, telematic communication, and continuous communication. These interventions were heterogeneous in terms of key components, including provider, timing, location, format, frequency, and duration. Not all studies provided detailed information on these elements. Telematic communication, particularly explanatory videos lasting at least 20 minutes in the waiting room, was found to be effective in significantly reducing family anxiety, as measured by the STAI scale.

**Conclusions:**

The results highlight the limited number of interventions designed to improve the experience of families and companions during surgical waiting periods. There is a clear need for standardized interventions and outcome measurement tools to optimize emotional support, ultimately improving the family experience and well-being in this context.

## INTRODUCCIÓN

La intervención quirúrgica comprende varias etapas, desde el ingreso del paciente al hospital, pasando por la cirugía en el bloque quirúrgico y su recuperación en la habitación, hasta el retorno al domicilio^1^^,^^2^. Este proceso, conocido como el periodo perioperatorio, es un momento crítico y potencialmente estresante tanto para pacientes como para sus familiares o acompañantes. Durante este periodo, los pacientes suelen experimentar ansiedad, incertidumbre y sensación de soledad, que pueden afectar negativamente su bienestar emocional y físico. Además, la naturaleza desconocida y a menudo impredecible de la cirugía intensifica estas reacciones emocionales^1^^,^^2^.

La familia o acompañantes, por su parte, a menudo viven este proceso de manera igualmente angustiante, considerando la situación no solo desde la perspectiva del paciente, sino también desde la de su propio bienestar. El estrés y la ansiedad que experimentan pueden impactar directamente en su capacidad cognitiva, dificultando la comprensión de las indicaciones proporcionadas por el personal sanitario. Esto puede repercutir en la calidad de los cuidados que brindan a la persona intervenida, al igual que en su capacidad para tomar decisiones informadas y colaborar de manera eficaz con el equipo médico^3^^-^^5^. Además, el ambiente hospitalario y la incertidumbre sobre el estado del paciente contribuyen a una percepción negativa de la experiencia hospitalaria, exacerbando la carga emocional tanto de pacientes como de sus familias y dificultando el proceso de adaptación al entorno hospitalario^3^^,^^6^^,^^7^

Esta situación se enmarca en el modelo de atención centrada en la familia en el contexto de la enfermería perioperatoria, enfoque que promueve un abordaje holístico hacia la familia durante el periodo perioperatorio, considerando tanto los factores mentales como los ambientales^8^. La evidencia demuestra que la familia desempeña un papel crucial en la adaptación del paciente a su nueva situación postquirúrgica^4^^,^^5^^,^^9^, hallazgo relevante porque las personas que cuentan con el apoyo de sus familiares tienden a obtener mejores resultados en su evolución postoperatoria, muestran mayor adherencia al tratamiento y desarrollan una actitud más positiva hacia el entorno hospitalario^7^.

Hasta la fecha se han publicado varias revisiones que examinan intervenciones destinadas a mejorar las experiencias de las familias o acompañantes de pacientes pediátricos durante el periodo perioperatorio^10^^-^^13^. Sin embargo, no se ha encontrado una revisión que aborde intervenciones focalizadas en la población adulta durante la espera quirúrgica hospitalaria. Esta falta de conocimiento representa una barrera para el diseño de estrategias efectivas y personalizadas que puedan abordar las necesidades específicas de las familias de adultos en este contexto. La implementación de dichas estrategias podría tener un impacto significativo, no solo en el bienestar y satisfacción de las familias, sino también en los resultados clínicos de los pacientes y en la eficiencia organizativa de los hospitales.

Por ello, se plantea esta revisión con el objetivo de identificar las intervenciones más efectivas para mejorar la experiencia de las familias o acompañantes durante la espera quirúrgica en el contexto hospitalario, proporcionando una base para desarrollar prácticas más centradas en las necesidades de los acompañantes y del sistema sanitario en su conjunto.

## MATERIAL Y MÉTODOS

### Diseño y estrategia de búsqueda

Se llevó a cabo una revisión sistemática en las bases de datos PubMed, CINAHL, SCOPUS, PsycINFO y Cochrane siguiendo la guía *Preferred Reporting Items for Systematic Reviews and Meta-Analyses* (PRISMA)^14^, de la literatura publicada entre enero de 2013 y diciembre de 2023. El protocolo de la revisión no ha sido publicado. La revisión se centró en responder a la siguiente pregunta de investigación ¿Cuáles son las intervenciones más efectivas para mejorar la experiencia de familiares o acompañantes durante la espera quirúrgica en el contexto hospitalario?, estructurada según el modelo PIO (población-intervención-resultados/outcomes):

- Población (P): Familiares o acompañantes de pacientes sometidos a procedimientos quirúrgicos.
- Intervención (I): Intervenciones de enfermería dirigidas a mejorar la experiencia de las familias durante la espera quirúrgica.
- Resultado (O): Mejora de la experiencia de las familias o acompañantes durante la espera quirúrgica, en términos de satisfacción, ansiedad y comprensión del proceso quirúrgico.

**Tabla 1:** Estrategias de búsqueda

Base de datos	Ecuación de búsqueda
Medline	(“Family”[MeSH Terms] OR “Caregivers”[All Fields] OR “Family members”[All Fields] OR “Relatives”[All Fields]) AND (((((“Nurses”[MeSH Terms] OR “Nursing interventions”[All Fields] OR “Nursing care”[MeSH Terms] OR “Nursing practice”[All Fields]) AND “Anxiety reduction”[All Fields]) OR “Family experience”[All Fields]) AND “Family anxiety”[All Fields]) OR “Caregiver anxiety”[All Fields] OR “Operative waiting time”[All Fields] OR “Surgical waiting time”[All Fields] OR “Waiting experience”[All Fields] OR “Perioperative stress”[All Fields])
Scopus	(“Family” OR “Caregivers” OR “Family members” OR “Relatives”) AND (((((“Nurses” OR “Nursing interventions” OR “Nursing care” OR “Nursing practice”) AND “Anxiety reduction”) OR “Family experience”) AND “Family anxiety”) OR “Caregiver anxiety” OR “Operative waiting time” OR “Surgical waiting time” OR “Waiting experience” OR “Perioperative stress”)
Pyscinfo	(“Family”[MM] OR “Caregivers”[All Fields] OR “Family members”[All Fields] OR “Relatives”[All Fields]) AND (((((“Nurses”[MM] OR “Nursing interventions”[All Fields] OR “Nursing care” OR “Nursing practice”[All Fields]) AND “Anxiety reduction”[All Fields]) OR “Family experience”[All Fields]) AND “Family anxiety”[All Fields]) OR “Caregiver anxiety”[All Fields] OR “Surgical waiting time”[All Fields] OR “Waiting experience”[All Fields] OR “Perioperative stress”[All Fields])
Biblioteca Cochrane	(“Family”[MeSH descriptor] OR “Caregivers”[All Fields] OR “Family members”[All Fields] OR “Relatives”[All Fields]) AND (((“Nurses”[MeSH descriptor] OR “Nursing interventions”[All Fields] OR “Nursing care”[MeSH descriptor] OR “Nursing practice”[All Fields]) AND “Anxiety reduction”[All Fields]) OR “Family experience”[All Fields] OR “Family anxiety”[All Fields] OR “Caregiver anxiety”[All Fields] OR “Operative waiting time”[All Fields] OR “Surgical waiting time”[All Fields] OR “Perioperative stress”[All Fields])
CINAHL	(“Family”[MM] OR “Caregivers”[All Fields] OR “Family members”[All Fields] OR “Relatives”[All Fields]) AND (((((“Nurses”[MM] OR “Nursing interventions”[All Fields] OR “Nursing care” OR “Nursing practice”[All Fields]) AND “Anxiety reduction”[All Fields]) OR “Family experience”[All Fields]) AND “Family anxiety”[All Fields]) OR “Caregiver anxiety”[All Fields] OR “Surgical waiting time”[All Fields] OR “Waiting experience”[All Fields] OR “Perioperative stress”[All Fields])

Las búsquedas fueron realizadas utilizando los términos: “*Family*”, “*Nursing interventions*”, “*Family experience*”, “*Operative waiting time*” y sus términos alternativos combinados con los operadores booleanos *AND* y *OR*. La estrategia de búsqueda, validada por un bibliotecario académico, fue pilotada en Medline (a través de PubMed) y posteriormente adaptada a las características de cada una de las bases de datos, tal como se muestra en la Tabla 1. La búsqueda se limitó a artículos publicados en los últimos 11 años (entre enero de 2013 y diciembre de 2024), en inglés o español. Los artículos recuperados se exportaron a *Microsoft Excel*, utilizando la función *Eliminar duplicados*.

Se cribaron los estudios que cumplían los siguientes criterios de inclusión: pacientes adultos mayores de 18 años sometidos a una intervención quirúrgica, e intervenciones enfermeras, realizadas con la familia/acompañantes durante el periodo perioperatorio para mejorar la experiencia de los familiares/acompañantes; se excluyeron intervenciones quirúrgicas urgentes y de cirugía menor ambulatoria. Se completó la búsqueda revisando las referencias bibliográficas de los estudios seleccionados.

### Síntesis y abstracción de los datos

Se diseñó una hoja de extracción estandarizada para los artículos incluidos, la cual fue completada manualmente para organizar y sistematizar la información. Se analizaron los resultados obtenidos de los estudios mediante varios criterios, siguiendo las recomendaciones del manual de Cochrane^15^. Se consideraron el país de origen y el año de realización, el objetivo del estudio, el diseño y el tamaño de la muestra. También se evaluaron las características específicas de las intervenciones, los instrumentos empleados para medir su efectividad, los principales resultados obtenidos y la calidad metodológica de cada estudio.

Inicialmente, cada investigadora analizó de manera individual las características de las intervenciones, análisis que fue posteriormente revisado y discutido por todo el equipo para alcanzar una síntesis integral de los resultados obtenidos. Los hallazgos clave de la revisión se presentaron en forma de tablas. El tamaño del efecto de las intervenciones fue calculado por una de las autoras (MVC) con el asesoramiento de una experta en estadística, utilizando una hoja de cálculo en Excel diseñada específicamente para obtener los valores d de Cohen. Este tamaño del efecto se interpretó de acuerdo con la convención de Cohen: tamaños cercanos a 0,2 se consideraron pequeños, entre 0,5 y 0,8 medianos, y superiores a 0,8 grandes.

### Evaluación de la calidad

La calidad metodológica de los estudios se evaluó utilizando las herramientas de lectura crítica del *Joanna Briggs Institute* (JBI)^16^. Cada ítem fue revisado por dos investigadoras (MJM y MVC), clasificando los estudios como de alta calidad si todas las respuestas eran *sí*, de calidad moderada si algún ítem generaba dudas o recibía una respuesta *no*, y de baja calidad si más de dos ítems tenían respuestas negativas.

Además, dos autoras (EMG y MVC) evaluaron el riesgo de sesgo en los ensayos clínicos aleatorizados (ECA) utilizando la herramienta Cochrane, que examina siete dominios clave que pueden influir en el riesgo de sesgo: 1) generación de la secuencia aleatoria, 2) ocultación de la asignación, 3) cegamiento de participantes y personal, 4) cegamiento de los evaluadores, 5) datos incompletos, 6) selectividad en los resultados e informes sesgados, y 7) otros errores. Según los criterios establecidos, los estudios fueron clasificados como de riesgo de sesgo bajo, alto o incierto. En caso de no disponer de suficiente información para una evaluación concluyente, se indicó que el riesgo de sesgo no podía determinarse con certeza.

## RESULTADOS

### Resultados de la búsqueda

**Figura 1:**
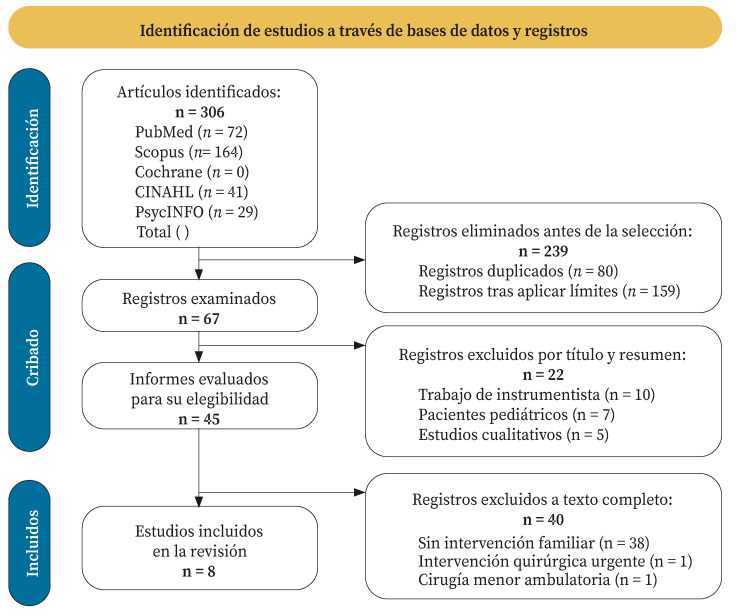
Diagrama de flujo PRISMA.

Inicialmente se identificaron 306 estudios, de los que se seleccionaron 67 tras eliminar duplicados y aplicar los límites de búsqueda. De estos se examinaron 45 a texto completo, y la aplicación de los criterios de selección permitió seleccionar ocho estudios que se incluyeron en la revisión^17^^-^^24^ (Fig. 1).

Las características de los estudios se muestran en la Tabla 2. Los estudios se llevaron mayoritariamente en Estados Unidos^17^^,^^18^^,^^22^ (37,5%) e Irán^19^^,^^23^ (25%); Brasil^24^, Australia^20^ y Corea del Sur^21^ contribuyeron con un estudio cada uno (12,5% cada uno). Seis eran estudios cuasi-experimentales (75%)^17^^-^^22^ y dos eran ECA^23^^,^^24^.

Los estudios revisados incluyeron 889 participantes, un 57,16% mujeres (descontando las 282 personas participantes de un estudio que no detalló la composición por sexo^18^). Solo cinco estudios^17^^,^^19^^,^^22^^-^^24^ especificaron la relación de familiares o acompañantes con los pacientes; el 63,26% eran familiares (n=384) y el resto amigos o personas cercanas al paciente, aunque en la mayoría de los casos no se indicó la relación concreta.

### Tipos de intervención

Todos los estudios proporcionaron información estandarizada y estructurada^17^^-^^24^ sobre aspectos relevantes como el funcionamiento del área quirúrgica o el tiempo estimado de la cirugía. Se identificaron tres tipos de intervenciones en torno a la comunicación destinadas a mejorar la experiencia de la familia durante la espera quirúrgica: estructurada, telemática y continuada.

Dos de los estudios^19^^,^^20^ optaron por la comunicación estructurada, proporcionando información a la familia de manera programada, ordenada y coherente. Esta intervención buscaba garantizar que la información fuese clara, comprensible y fácilmente asimilable por los receptores e incluyó detalles sobre el procedimiento quirúrgico, los tiempos estimados, los pasos a seguir antes y después de la cirugía, así como los posibles resultados^19^^,^^20^. El enfoque estructurado tenía como objetivo evitar la confusión y facilitar una comprensión completa y ordenada del proceso quirúrgico, lo que contribuyó a reducir la ansiedad y la incertidumbre entre los familiares^19^^,^^20^.

Cuatro estudios eligieron la comunicación telemática^18^^,^^21^^,^^23^^,^^24^, incorporando medios electrónicos como mensajes, vídeos u otras herramientas digitales, como aplicaciones móviles y plataformas web, para mejorar el flujo de información relevante y facilitar la transmisión eficiente a la familia de actualizaciones en tiempo real sobre el progreso del procedimiento quirúrgico.

Los dos estudios restantes^17^^,^^22^ emplearon la comunicación continuada posibilitando una comunicación fluida y constante durante el periodo perioperatorio. Este enfoque implicó la designación de una enfermera de enlace cuyas responsabilidades abarcaron responder a las preguntas de los familiares, proporcionarles apoyo emocional durante la espera quirúrgica y brindar actualizaciones periódicas y relevantes sobre el progreso de la cirugía^17^^,^^22^. Además de transmitir información y actuar de intermediaria entre familia y equipo médico, ser un recurso accesible y dedicado contribuyó a establecer una conexión más estrecha y personalizada que fomento un ambiente de confianza y comprensión mutua, proporcionando un apoyo integral a la familia y mejorando su ansiedad durante la espera quirúrgica^17^^,^^22^.

### Componentes de las intervenciones

El tipo de cirugía varió entre estudios, influyendo con sus particularidades y demandas específicas en las intervenciones realizadas: cirugía general^21^^-^^23^, ortopédica^18^^,^^20^, vascular^19^^,^^24^ y oncológica^17^.

A pesar de esa variabilidad, se identificaron componentes comunes a las distintas intervenciones, como el proveedor y el receptor de la misma y, en relación a la comunicación, el formato utilizado, el momento y el lugar, y la frecuencia o duración de la misma (Tabla 2).

*Proveedores*. Los proveedores de las intervenciones analizadas mostraron una considerable variabilidad. Cuatro estudios^17^^,^^20^^-^^22^ identificaron a las enfermeras como las principales facilitadoras de la información. Dos de ellos especificaron que el proveedor de la información era una enfermera de enlace^17^^,^^22^; uno detalló la formación académica requerida (poseer un máster de especialidad, ser especialista en clínica, o contar con un diploma en cirugía médica)^17^, mientras que el otro no especificó la formación académica, pero describió sus funciones (gestionar la sala de espera, facilitar la interacción entre el personal médico y la familia, y asistir a la primera visita postcirugía)^22^. En los dos ECA, la información fue proporcionada por el investigador^23^^,^^24^, siendo uno de ellos enfermero^24^. En el estudio de Tagadaya y col^18^ la información fue facilitada por el personal de la sala de espera, lo que genera ambigüedad al no especificar su categoría (personal celador, auxiliar de enfermería, o enfermería). Un estudio no precisó quién fue el proveedor de la información^19^.

*Receptores*. Aunque la mayoría de intervenciones identificadas se orientaron hacia familiares o acompañantes que se encontraban en la sala de espera^18^^-^^21^^,^^23^^,^^24^, dos estudios identificaron la figura de un cuidador principal que actuaba como el canal principal para recibir y comunicar toda la información relativa al paciente que estaba siendo intervenido^17^^,^^22^.

*Formato*. La mayoría de estudios entregaron documentación escrita, como las tarjetas informativas^1^^-^^7^^,^^19^^,^^20^^,^^22^, combinándolas en dos estudios con comunicación verbal^17^^,^^22^. Otros estudios utilizaron medios técnicos, como vídeos^23^^,^^24^ o mensajes de texto^21^.

*Momento y frecuencia.* La planificación de la comunicación en momentos específicos, con variaciones entre estudios, aseguró una entrega consistente de información. En los dos estudios cuya intervención fue liderada por una enfermera de enlace^17^^,^^22^, mantuvo contacto con la familia cada dos horas durante todo el proceso perioperatorio. Un estudio^23^ realizó dos intervenciones (al inicio y durante la cirugía) de 30-40 minutos; el resto implementó una única intervención, ya sea antes de la cirugía^19^^,^^24^ o durante la misma^18^, con un tiempo estimado de veinte a treinta minutos^23^ o no especificado^18^^,^^19^.

### Instrumentos utilizados para evaluar las intervenciones

Se emplearon distintos tipos de instrumentos para evaluar las intervenciones. Un estudio realizó una encuesta *ad-hoc*^17^ para conocer la percepción de los familiares sobre la información recibida (cómo, cuándo, utilidad, nivel de estrés posterior y apoyo emocional recibido). Otro estudio midió el nivel de satisfacción mediante el cuestionario *Consumer Assessment of Health Prioviders and Systems* (CAHPS). El resto midieron el nivel de ansiedad, bien mediante una escala (Escala Analógica Visual, EAV)^18^, bien con el Inventario Ansiedad Estado de Spielberger (IDATE)^19^^,^^24^ o con el Inventario de Ansiedad Estado-Rasgo de Spielberg (STAI)^20^^,^^21^^,^^23^.

### Efectividad de las intervenciones

Dada la heterogeneidad de las medidas de resultados utilizadas en los estudios revisados, no fue posible determinar qué intervención fue la más efectiva para mejorar la experiencia de las familias durante la espera quirúrgica. La comunicación telemática mediante un vídeo explicativo de al menos 20 minutos, antes y durante la cirugía en la sala de espera, redujo significativamente la ansiedad de los familiares, medida en dos ECA con los inventarios STAI^23^ e IDATE^24^, con tamaños del efecto grande y muy grande respectivamente. Asimismo, la entrega de una tarjeta informativa en la sala de espera antes de la cirugía mejoró de forma significativa la ansiedad de 118 familias, medida con el inventario IDATE, con un tamaño de efecto medio^19^ (Tabla 2).

**Tabla 2:** Estudios incluidos en la revisión

Autor	Diseño	Resultados principales
País	Muestra	Tamaño del efecto (d de Cohen)
Año	Intervención (tipo y componentes)	
	Instrumento de evaluación	
Tagadaya y col^18^	EC	↓ similar del nivel de ansiedad de familiares en el GI (9,89; DE=6,42) con respecto al GC (9,92; DE=6,76)
EEUU	n = 120	dM= 0,03 (IC95%:-3,76 a -3,70); p=0.997
2013	Comunicación telemática con uso de busca personas	No aplicable
	- proveedor: n/e	
	- momento: durante	
	- lugar: sala de espera	
	- formato: buscapersonas/en persona	
	- frecuencia: n/e	
	- duración: n/e	
	EAV	

Herdy col^22^	EC	↑ la satisfacción un 9% en las familias (p=0,03)
EEUU	n = 60	
2014	Comunicación continuada con enfermera de enlace	No aplicable
	- proveedor: enfermera de enlace	
	- momento: antes, durante y después	
	- lugar: n/e	
	- formato: tablero electrónico, tarjeta informativa y en persona	
	- frecuencia: cada 2 horas	
	- duración: n/e	
	CAHPS	

Hanson-Heath	CE	↑ el contacto con las familias de un 77% a un 98%
y col^17^	n=102	96% consideró útil la información
EEUU	Comunicación continuada con enfermera de enlace	88% disminuyó su ansiedad
2016	- proveedor: enfermera de enlace	81% prefirió la comunicación en persona a la telefónica
	- momento: antes, durante y después	No aplicable
	- lugar: n/e	
	- formato: tarjeta informativa, en persona y por teléfono	
	- frecuencia: cada 2 horas	
	- duración: n/e	
	Encuesta *ad-hoc*.	

Azarfarin y col ^19^	EC	↓ significativa de la ansiedad de familiares en el
Irán	n=118 (GI = 59; GC = 59)	GI (45,9; DE=12,2) respecto al GC (51,7; DE=13,4)
2016	Comunicación estructurada con tarjeta informativa	dM=5,8 (IC95%:-10,42 a -1,18); p=0,0016
	- proveedor: n/e	d de Cohen: 0,45
	- momento: antes	
	- lugar: sala de espera	
	- formato: tarjeta informativa	
	- frecuencia: 1 vez	
	- duración: n/e	
	IDATE	

Hamester y col^24^	ECA	↓ significativa de la ansiedad de familiares en el
Brasil	n=210 (GC= 105; GI= 105)	GI (41,3; DE=8,6) respecto del GC (50,6; DE=9,4)
2016	Comunicación telemática con vídeo	dM=9,3 (IC95%: 6,8-11,7); p<0,001
	- proveedor: investigador enfermera	d de Cohen: 1,03
	- momento: antes	
	- lugar: sala de espera	
	- formato: vídeo	
	- frecuencia: n/e	
	- duración: 20 minutos	
	IDATE	

Kynoch y col^20^	EC	Diferencias ns entre GI (35,03; DE=11,07)
Australia	n= 129 (GI= 63; GC= 66)	y GC (36,85; DE= 12,51)
2017	Comunicación estructurada con tarjeta informativa	dM=-1,82 (IC95%: -4,7 a 4,7); p=0,573
	- proveedor: enfermera	d de Cohen: 0,15
	- momento: antes, durante y después	
	- lugar: sala de espera	
	- formato: tarjeta informativa	
	- frecuencia: n/e	
	- duración: n/e	
	STAI	
Mi y Vasuki^21^	EC	↓ ns del nivel de ansiedad en el GI pre-post intervencion (46,42; DE=11,52 vs 54,50; DE=9,43)
Corea del Sur	n= 48 (GI=24; GC=24)	dM = -8,08 (IC95%: -9,00 a 1,20); p=0,143
2017	Comunicación telemática del progreso	Sin diferencias pre-post en el nivel de ansiedad
	de la cirugía vía SMS	del GC (52,25; DE=11,64 vs 52,67; DE=8,78)
	- proveedor: enfermera	dM= +0,42 (IC95%: -3,18 a 4,02); p=0,572
	- momento: durante	Diferencias post ns entre GI (46,42; DE=11,52)
	- lugar: sala de espera	y GC (52,67; DE=8,78)
	- formato: SMS	dM=-6,25 (IC95%: -9,50 a 1,00); p= 0,456
	- frecuencia: 3 veces	d de Cohen: 0,77
	- duración: no se especifica	
	STAI	

Bagheri y col^23^	ECA	↓ significativa de la ansiedad pre- post intervención en GI:
Irán	n=102 (GC= 34; GI1= 34; GI2= 34)	GI1 (video) ↓ de 47,96 (DE=7,44) a 43,00 (DE=4,16)
2022	Comunicación telemática con vídeo (GI1) e informe de progreso intraoperatorio GI2)	dM = -4,96 (IC95%: -8,14 a -1,78) p<0,001
	- proveedor: investigador	GI2 (informe) ↓ de 45,14 (DE=4,58) a 42,47 (DE=3,68)
	- momento: antes y durante	dM = -2,67 (IC95%: -4,86 a -0,48); p<0,001
	- lugar: sala de espera	GC ↑ansiedad ns pre- postintervención (42,41;
	- formato: vídeo	DE=6,09 vs 42,52; DE=5,50)
	- frecuencia: no se especifica	dM = +0,11 (IC95%: -2,95 a 3,17); p=0,850
	- duración: 30/40 minutos	dM (GI1-GC): -5.0 (IC95%: -8,30 a -1.84); p=0,0031
	STAI	dM (GI2-GC): -2.78 (IC95%:-5,34 a -0,22; p=0,037
		d de Cohen: 0,82

CAHPS: *Consumer Assessment of Health Plans Stud providers and systems*; dM: diferencia de medias; DE: Desviación estándar; EAV: Escala analógica visual; EC: Estudio cuasi-experimental; ECA: ensayo clínico aleatorizado; GI: Grupo Intervención; GC: Grupo Control;IC: intervalo de confianza; IDATE: Inventario Ansiedad Estado de Spielberg; n/e: no se especifica; ns: no significativo; SMS: mensaje de texto; STAI: Inventario de Ansiedad Estado-Rasgo de Spielberg.

La calidad de los seis estudios cuasi-experimentales^17^^-^^22^ fue moderada-alta (puntuaciones entre 6 y 9 sobre 9) y la calidad de los ECA^23^^,^^24^ fue moderada (puntuación 9 sobre 13) (Tabla 3).

**Tabla 3:** Calidad metodológica de la calidad de los estudios incluidos en la revisión mediante la *Joanna Briggs Institute Critical appraisal Checklist*^16^

Estudio	Ítems													*Score*
	1	2	3	4	5	6	7	8	9	10	11	12	13	
*Estudios cuasi-experimentales*														*/9*
Hanso-Health y col^17^	S	S	N	S	N	N	S	S	S					6
Tagadaya y col^18^	S	S	S	S	S	S	S	S	S					9
Azarfarin y col^19^	S	S	S	S	S	S	S	S	S					9
Kynoch y col^20^	S	S	S	S	N	N	S	S	S					7
Mi y Vasuki^21^	S	S	S	S	N	D	S	S	S					7
Herd y col^22^	S	S	S	S	D	S	S	S	S					8
*Estudios clínicos aleatorizados*														*/13*
Bagheri y col^23^	S	D	S	D	D	S	S	S	S	D	S	S	S	9
Hamester y col^24^	S	D	S	N	N	S	N	S	S	S	S	S	S	9

S: sí; N: no; D: dudoso.

Estudios cuasi-experimentales: 1. ¿Está claro en el estudio cuál es la causa y cuál es el efecto en relación a las variables? 2. ¿Fueron similares los participantes incluidos en alguna comparación? 3. ¿Se incluyeron los participantes en alguna comparación que recibiera un tratamiento/cuidado similar aparte de la exposición o intervención de interés? 4. ¿Hubo un grupo de control? 5. ¿Hubo múltiples mediciones del resultado antes y después de la intervención/exposición? 6. ¿Se completó el seguimiento y, de no ser así, se describieron y analizaron adecuadamente las diferencias entre los grupos en cuanto a su seguimiento? 7. ¿Se midieron de la misma manera los resultados de los participantes incluidos en alguna comparación? 8. ¿Fueron los resultados analizados en una forma estandarizada, válida y veraz para casos y controles? 9. ¿Fue el tiempo de exposición al evento lo suficientemente largo como para ser significativo?

Estudios clínicos aleatorizados: 1. ¿Se asignaron los participantes mediante una aleatorización verdadera en el grupo de intervención? 2. ¿Se ocultó la asignación de grupos? 3. ¿Eran similares los grupos al inicio del estudio? 4. ¿Se les ocultó a los participantes la asignación de los tratamientos? 5. ¿Desconocían aquellos administrando el tratamiento y la asignación de los participantes? 6. ¿Se trató de forma idéntica a los grupos de tratamiento aparte de la intervención de interés? 7. ¿Los evaluadores de resultado estaban cegados a la asignación del tratamiento? 8. ¿Se midieron los resultados de la misma manera para los grupos de tratamiento? 9. ¿Se midieron los resultados de forma fiable? 10. ¿Se completó el seguimiento y, de no ser así, se descubrieron y analizaron adecuadamente las diferencias entre grupos en cuanto a su seguimiento? 11. ¿Se analizaron los participantes en los grupos a los que fueron asignados al azar? 12. ¿Se utilizó un análisis estadístico adecuado? 13. ¿Fue apropiado el diseño del ensayo y se tuvo en cuenta cualquier desviación del diseño estándar del ECA (aleatorización individual, grupos paralelos) en la realización y el análisis del ensayo?

**Tabla 4:** Evaluación de riesgo de sesgo de los estudios clínicos aleatorizados incluidos en la revisión

	Bagheri y col ^23^	Hamester y col ^24^
	2022	2016
Generación aleatoria de la secuencia (sesgo de selección)	Alto	Alto
Asignación oculta (sesgo de selección)	Bajo	Bajo
Cegado de participantes y personal (sesgo de ejecución)	Bajo	Bajo
Evaluación ciega de resultados (sesgo de detección)	Alto	Bajo
Datos incompletos de los resultados (sesgo de retirada)	Alto	Alto
Información selectiva de resultados (sesgo de información)	Alto	Alto
Otros errores	Alto	Alto

Ambos ECA presentaron un riesgo bajo en dos dominios y alto en cuatro o cinco de los dominios restantes, sin que se identificaran dominios con resultados desconocidos (Tabla 4).

## DISCUSIÓN

Esta revisión evidencia la escasez de intervenciones diseñadas para mejorar la experiencia de la familia durante la espera quirúrgica, sus componentes y efectividad. La heterogeneidad de las intervenciones, en términos de proveedor, momento, lugar, formato, duración e instrumentos de medida, complica la determinación de su efectividad. No obstante, el análisis realizado por una investigadora (MVC) ha permitido extraer conclusiones, identificar lagunas conceptuales y metodológicas y formular recomendaciones para futuros estudios.

Los resultados destacan el impacto significativo de la comunicación telemática en la disminución de la ansiedad familiar, según el análisis comparativo del tamaño del efecto realizado de los resultados de ansiedad evaluados con los inventarios STAI e IDATE. Ambos instrumentos mostraron una excelente fiabilidad, con coeficientes alfa de Cronbach superiores a 0,80, lo que asegura la consistencia interna en la medición de la ansiedad^25^. Específicamente, la comunicación telemática facilitada mediante un vídeo explicativo de al menos 20 minutos en la sala de espera, reduce significativamente tanto la ansiedad de estado (temporal) como la de rasgo (crónica) en las familias^26^. Este hallazgo puede atribuirse a varios factores clave: permite una entrega precisa, directa y detallada de información sobre el proceso quirúrgico y cuidados posteriores, lo que tranquiliza a las familias al mejorar su comprensión y preparación^18^^,^^21^^,^^23^^,^^24^. Además, el contacto visual y auditivo del vídeo puede establecer una conexión emocional y aumentar la confianza entre el personal sanitario y las familias, tal y como ocurre con los pacientes^26^, reduciendo así los niveles generales de ansiedad y mejorando la experiencia sanitaria percibida^22^^,^^23^^,^^27^.

Estos hallazgos son congruentes con investigaciones previas sobre el uso de tecnologías emergentes en el ámbito de la salud. En una reciente revisión sistemática se han identificado diecinueve indicadores de enfermería influidos por las Tecnologías de la Información y la Comunicación (TICs), abarcando aspectos como la comunicación y coordinación de cuidados, el tiempo dedicado a pacientes, y la accesibilidad y calidad de la comunicación^28^. Además, diversos estudios^7^^,^^28^^,^^29^ han subrayado la importancia de profundizar en la implementación de las TICs para optimizar la comunicación y atención en salud, enfatizando su rentabilidad y la capacidad de identificar y centrarse en las necesidades específicas de pacientes y familiares^30^.

Los resultados de algunos estudios incluidos en la revisión no especifican el proveedor de la información^18^^,^^19^^,^^23^^,^^24^, el modo específico de comunicación^(18,19,22,24^) o quién facilita la información escrita^19^^,^^20^^,^^24^, lo que dificulta replicar la intervención en otros contextos. Además, no se detallan adecuadamente características del receptor de la información, como la edad o el uso habitual de la tecnología, aspectos relevantes que pueden influir en la habilidad para utilizar las TICs y comprender la información facilitada^17^^-^^24^. La naturaleza del proveedor de la información también puede afectar la percepción y aceptación de la comunicación por los pacientes y sus familias^22^. La ambigüedad en estos puntos limita la aplicabilidad de los resultados y sugiere la necesidad de una definición más precisa de los componentes de la intervención en estudios futuros^31^.

Un estudio cuasi-experimental con 118 familias observó que la comunicación estructurada facilitada mediante una tarjeta informativa en la sala de espera mejora significativamente la ansiedad familiar^19^. Esto destaca la importancia de la claridad y accesibilidad de la información en entornos quirúrgicos para aliviar la ansiedad, sugiriendo que intervenciones simples y económicas, como las tarjetas informativas, pueden impactar notablemente en el bienestar emocional de las familias. A pesar de la buena calidad del estudio de acuerdo a los criterios del JBI, la naturaleza de su diseño implica que se debe tener cautela al generalizar estos resultados. Para confirmar estos efectos, sería beneficioso que los futuros estudios tuvieran diseños más robustos, como ECA^32^.

Un elemento común en todos los estudios revisados^17^^-^^19^^,^^21^^-^^24^ es la comunicación cara a cara, aunque su implementación no se ha explorado en profundidad debido a la falta de detalles precisos. Esto coincide con estudios cualitativos^33^^,^^34^ que identifican cinco aspectos clave de la espera quirúrgica: el entorno, las actividades realizadas, la comunicación, las expectativas y los sentimientos. La comunicación, ya sea mediante métodos pasivos como tableros electrónicos o interacciones presenciales, juega un papel fundamental en la reducción de la ansiedad, especialmente mediante palabras de aliento y consuelo^33^.

Es fundamental considerar la atención centrada en la familia en el contexto de la enfermería perioperatoria^8^, lo que implica reconocer a la familia como un componente integral del proceso quirúrgico y asegurar que sus necesidades y preocupaciones sean abordadas. La enfermería perioperatoria puede aplicar este enfoque al proporcionar información clara, apoyo emocional y facilitar la comunicación entre la familia y el equipo médico^34^. Un enfoque centrado en la familia no solo mejora la experiencia de los pacientes, sino que también puede contribuir a una recuperación más efectiva y a un mejor manejo de la ansiedad^8^^,^^34^.

Un área prometedora para futuras investigaciones es la efectividad de la comunicación híbrida (en persona y telemática) para reducir la ansiedad familiar durante la espera quirúrgica. Aunque las intervenciones telemáticas tienen un gran potencial, se ha manifestado preocupación sobre su impacto en la relación y comunicación entre el personal sanitario y las familias, especialmente en momentos críticos^30^. Algunos estudios cuasiexperimentales han mostrado resultados positivos de la comunicación híbrida en la disminución de la ansiedad familiar^17^^,^^18^^,^^22^ pero la variabilidad en los instrumentos de medición limita las comparaciones. Para avanzar en este campo, es esencial realizar ECA más robustos con grupos de control, utilizando instrumentos de medición consistentes para evaluar la ansiedad y comparar la eficacia de la comunicación híbrida frente a la telemática.

Esta revisión no está exenta de limitaciones. Los resultados se basan en una búsqueda restringida a las principales bases de datos de los últimos diez años y varios idiomas específicos, lo que pudo excluir estudios relevantes. El número reducido de estudios incluidos limita la capacidad para identificar intervenciones efectivas. Además, la mayoría de los estudios provienen de países como EEUU, Irán y Corea del Sur, lo que puede no reflejar la diversidad global y omitir intervenciones de otros países, afectando a la generalización de los resultados. A pesar de ello, el estudio se benefició de un riguroso proceso de revisión por pares, el uso de diversas fuentes en la estrategia de búsqueda, y el cumplimiento de las directrices.

En conclusión, esta revisión sistemática destaca la prometedora efectividad de la comunicación telemática mediante vídeos explicativos para mitigar la ansiedad de las familias durante la espera quirúrgica. A pesar de las limitaciones metodológicas y el número reducido de estudios disponibles, los hallazgos subrayan la importancia de estandarizar la descripción de intervenciones futuras (tipo de comunicación, proveedor, momento, lugar, formato, frecuencia y duración de las intervenciones) y las medidas de resultados utilizadas para evaluar la ansiedad. Estos pasos son esenciales para avanzar en la investigación y mejorar la comparabilidad entre estudios, facilitando así una evaluación precisa y generalizable de las estrategias diseñadas para apoyar emocionalmente a las familias en entornos quirúrgicos.

## Data Availability

Se encuentran disponibles bajo petición al autor de correspondencia.
